# Presence of neutralizing SARS‐CoV‐2 antibodies in asymptomatic population of N'Djamena, Chad

**DOI:** 10.1002/iid3.1154

**Published:** 2024-01-16

**Authors:** Andrillene Laure Deutou Wondeu, Mahamat Fayiz Abakar, Federica Frasca, Aleyo Zita Nodjikouambaye, Fatima Abdelrazakh, Nathan Naibei, Guy Rodrigue Takoudjou Dzomo, Djallaye Djimtoibaye, Joseph Mad‐Toingue, Carolina Scagnolari, Guido Antonelli, Giulia Linardos, Cristina Russo, Carlo Federico Perno, Fissou Henry Yandai, Sabrina Atturo, John Hiscott, Vittorio Colizzi, Giulia Cappelli, Nambatibe Ngueadoum, Alsadick Haroun, Ouchemi Choua, Ali Mahamat Moussa

**Affiliations:** ^1^ Laboratoire des Grandes Epidémies Tropicales (LAGET) Complexe Hospitalo‐Universitaire le Bon Samaritain N'Djamena Chad; ^2^ Department of Biology and Interdepartmental Centre for Comparative Medicine University of Rome Tor Vergata Rome Italy; ^3^ Laboratory of Molecular Biology and Immunopathology Evangelical University of Cameroon Mbouo‐Bandjoun Cameroon; ^4^ Institut de Recherche en Elevage pour le Développement (IRED) N'Djamena Chad; ^5^ Laboratory of Virology, Department of Molecular Medicine Sapienza University of Rome Italy; ^6^ Communauté des Amis de l'Informatique pour le Développement (CAID‐Tchad) N'Djamena Chad; ^7^ Centre Hospitalier Universitaire de Référence Nationale (CHU‐RN) N'Djamena Chad; ^8^ Coordination nationale de riposte sanitaire contre la Covid‐19, Ministère de la Santé Publique N'Djamena Chad; ^9^ Virology and Mycobacteriology Unit, “Bambino Gesù” Children Hospital—Healthcare and Research Institute—Rome Rome Italy; ^10^ Institute Pasteur Cenci‐Bolognetti Foundation Rome Italy; ^11^ Institute for Biological Systems National Research Council Rome Italy; ^12^ Direction Générale des Laboratoires, Pharmacie & Médicaments, Ministère de la Santé Publique N'Djamena Chad

**Keywords:** anti‐receptor‐binding domain antibodies, asymptomatic individuals, N'Djamena, neutralizing antibodies

## Abstract

**Introduction:**

Neutralizing antibodies (NAbs) are an important specific defence against viral infections, as these antibodies bind to specific receptor(s) and block the viral entry. NAbs assessments are therefore useful in determining individual or herd immunity to SARS‐CoV‐2. This study aims to deepen the investigation by assessing the positivity rate of neutralizing anti‐spike antibodies to understand the real protection of the studied population against SARS‐CoV‐2.

**Methods:**

This study involved 260 plasma samples from a larger cohort of 2,700 asymptomatic volunteer donors, enrolled between August and October 2021 in health facilities of N'Djamena. In this study four different kits and techniques including the pseudotype assay have been used and compared with detect the SARS‐CoV‐2 antibodies. Pseudotyped vesicular stomatitis virus (VSV), was used both the identify and measure the NAbs that to evaluate the performance of two cheaper and easy to use commercial kits, specific for the detection of receptor‐binding domain antibodies (anti‐RBD) against the SARS‐CoV‐2 spike protein.

**Results:**

The VSV spike neutralization assay showed that 59.0% (*n* = 59) samples were positive for NAbs with titers ranging from 1:10 to 1:4800. While 23 out the 41 negative NAbs samples were detected positive using anti‐RBD (Abbott) test. Furthermore, a direct and significant strong correlation was found between NAbs and anti‐RBD, specifically with Abbott kit. Taken together, the Roche and Abbott methods indicated agreement at the high concentrations of antibodies with the VSV‐pseudovirus method. Abbott and Roche indicated a good sensitivity, but the Abbott system test appeared to have better specificity than the Roche test.

**Conclusion:**

Our findings indicated a high presence of NAbs against SARS‐CoV‐2 spike protein among asymptomatic individuals in N'Djamena. This could be one of the reasons for the low severity of Covid‐19 observed in this area, given the key role of NAbs in blocking SARS‐CoV‐2 infection.

## Graphical abstract

The high presence of neutralizing antibodies against the SARS‐CoV‐2 spike protein among asymptomatic populations in N'Djamena could explain the low prevalence and low severity of Covid‐19 observed in these areas, as these neutralizing antibodies play a key role in blocking SARS‐Cov‐2 infection. Moreover, the commercial tests available (Abbott Alinity i and Roche Elecsys®) for the detection of these receptor‐binding domain antibodies (anti‐RBDs) showed acceptable performance when compared with the pseudovirus neutralization test.

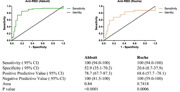

## 1 INTRODUCTION

N'Djamena, the capital city of Chad, is the city that has reported the most cases of Covid‐19 since the first case notified in Chad by the government on March 19, 2020. World Health Organization (WHO) data indicate that Chad is one of the countries in Central Africa with the lowest number of reported cases and deaths due to Covid‐19 (7698 confirmed cases with 194 deaths).^1^ These number in African counties could be underestimated due to the difficulties and limitations of molecular testing^2^ which makes the seroprevalence studies, carried out in these countries, of great importance. Indeed some seroprevalence studies conducted in this region of Africa have shown a high circulation of the virus in the asymptomatic population.^3^ However, the majority of these studies have been conducted using cheaper commercial kits that detect nucleocapsid proteins, which are not specific for SARS‐CoV‐2 virus. Indeed, these proteins show significant sequence conservation with other coronavirus nucleocapsid proteins.^4^, ^5^ This cross reaction could explain the high anti‐N seroprevalence observed in these countries. Moreover, it is conceivable that SARS‐CoV‐2 has circulated in asymptomatic way in African countries.

To give support to this hypothesis it is necessary to investigate the immune response against the spike surface protein. The SARS‐CoV‐2 spike protein, formed by two subunits (S1 and S2), plays a role in binding to receptors and facilitating membrane fusion. The S1 subunit binds the virions to the cell membrane, through its interaction with ACE‐2 receptor, while S2 facilitates fusion between virions and cell membranes.^6^ Spike‐specific antibody response plays a major role in the protection against SARS‐CoV‐2 entrance and infection.^7^ Human neutralizing antibodies (NAbs) are specific for epitopes on the viral surface that mediate viral entry into a host cell through, the receptor. NAbs bind the epitopes located on spike protein blocking the ACE2 receptor binding domain (RBD).^8^, ^9^ Unlike to anti‐nucleocapsid antibodies, the presence of NAbs would be a good indicator of protective immunity against SARS‐CoV‐2 infection, as it is the case for other viral infections.^10^ A serological assay capable of directly measuring NAbs would therefore be preferable to those serological tests which detect nonspecific spike binding antibodies.^7^

Many approaches were developed to detect NAbs against SARS‐CoV‐2, such as, enzyme‐linked immunosorbent assay (ELISA), rapid lateral flow assay, microneutralization assay and SARS‐CoV‐2 pseudotyped virus assay.^11^ However, the current gold standard for the detection of NAbs is the viral neutralization test, which cannot be routinely performed in clinical laboratories, as it requires the use of live virus and a BSL3 containment facility. Alternative SARS‐CoV‐2 pseudovirus technology uses lentiviral particles based on human immunodeficiency virus, retroviral particles based on murine leukemia virus, or systems based on vesicular stomatitis virus (VSV), have been developed to avoid the culture of live SARS‐CoV‐2. The results from such pseudovirus neutralization assays correlate well with the results of measurements using authentic viruses.^12^, ^13^, ^14^ Although alternative BSL2 protocols using pseudotyped have been developed, these methods remain in the research area.^15^

Since the beginning of the Covid‐19 pandemic, several highly transmissible variants of SARS‐CoV‐2 have emerged.^16^, ^17^ Some mutations in the SARS‐CoV‐2 spike protein (S) can alter antigenic properties, allowing them to be more transmissible, virulent, pathogenic or evade immunity induced by previous infection.^12^ In this situation, it would be important to consider these mutations in the production process of pseudoviruses to assess the efficacy of NAb production in vaccines, patients, and asymptomatic individuals.

Several serological tests are available for the detection of anti‐SARS‐CoV‐2 antibodies in routine diagnosis in clinical laboratories, but the challenge is to know which one to use according to the objectives of the study, the cost, and the context.^18^ In this study, we assessed the humoral immune response to SARS‐CoV‐2 by detecting NAbs in asymptomatic individuals. The conventional method used allowed to evaluate the performance of commercial serological tests.

## 2 MATERIALS AND METHODS

### 2.1 Description of the study site

N'Djamena, the political capital and largest city of Chad, with an estimated population of 1,533,000 in 2022.^19^ It lies directly on the border with the far north of Cameroon, at the confluence of the Chari and Logone rivers. Composed of 10 districts, with a hot semi‐arid Sahelian climate, N'Djamena is one of the cities reporting a high prevalence of Covid‐19 in Chad.^1^, ^20^

### 2.2 Study design

Samples were selected from voluntary donors attending routine consultations in the health facilities, between August and October 2021.

Given that in the previous study,^21^ on the aforementioned cohort the SARS‐CoV‐2 positive rate, using IgG antibodies against SARSCoV‐2 nucleocapsid protein ELISA kits, was 69.5%, we focused our study on the identification of the presence of NAbs in a selected seropositive group of this population. Considering the role of such antibodies in conferring potential protection to Covid‐19 and to future possible coronavirus‐related diseases, we compared in this study four different kits including the pseudotype assay, which is the gold standard for NAbs detection. According to the previous study^21^ the selected 260 samples were distributed as follows: anti‐N positive (210 samples), anti‐N equivocal (28 samples). A group of negative anti‐N samples (22 samples) was included as a control.

**Figure 1:**
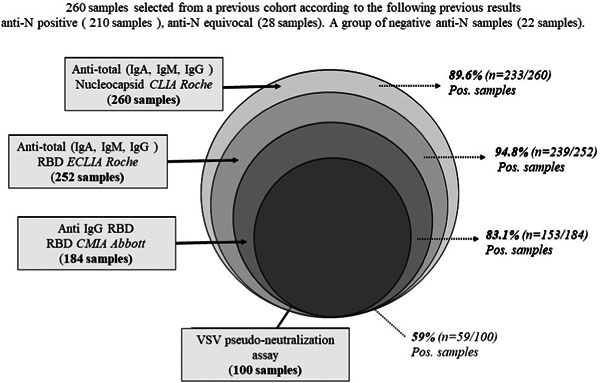
Flowchart describing the study design process. Anti‐total N, IgG, IgM, IgA antibodies against SARS‐CoV‐2 nucleocapsid; Anti total RBD, IgG, IgM, IgA antibodies against SARS‐CoV‐2 receptor binding domain; Anti IgG RBD, IgG antibodies against SARS‐CoV‐2 receptor binding domain; CLIA, chemiluminescence assay; CMIA, chemiluminescent microparticle immunoassay; ECLIA, electro‐chemiluminescence immunoassay; VSV pseudo‐neutralization assay, vesicular stomatitis virus pseudotyped virus‐based neutralization assay.

Below are the investigations carried out (Figure 1):

(i) All of these samples were analyzed for the determination of total anti‐N antibodies against nucleocapsid protein of SARS‐CoV‐2, by qualitative detection test, using chemiluminescence assay (CLIA) Elecsys® Anti‐SARS‐CoV‐2 N (Roche).
(ii) The determination of total anti‐SARS‐CoV‐2 antibodies directed against the spike protein was carried out with of 252 of 260 plasmas, analyzed by the Electro‐chemiluminescence immunoassay (ECLIA) double‐antigen sandwich method by using Elecsys® Anti‐SARS‐CoV‐2 S assay (Roche).
(iii) The detection of anti‐IgG SARS‐CoV‐2 antibodies against RBD was performed with 184 plasmas, the test was conducted according to the recommendations of the manufacturer Abbott.
(iv) A total of 100 samples were analyzed for the characterization of anti‐Spike NAbs using pseudotyped VSV.

The difference in number of individual tested among the different kits is due to the availability of biological samples (plasma) and to the cost of single analysis.

### 2.3 Anti‐nucleocapsid protein (anti‐N) detection

The determination of SARS‐CoV‐2 anti‐N antibodies to the nucleocapsid antigen, was performed in the Virology and Immunology Laboratory of the Bambino Gesù Hospital in Rome. Chemiluminescence assay (CLIA) was used to identify total antibodies (IgA, IgM, or IgG) directed against the N protein of the SARS‐CoV‐2. This was the qualitative detection test.

The assays were performed according to the manufacturer's recommendations of Elecsys® Anti SARS‐CoV‐2 immunoassay (Roche Diagnostics, CH) based on the sandwich principle. The sensitivity reported by the manufacturer was 99.5% and the specificity 99.8%. Result was considered anti‐N positive with cut‐off index ≥1.

### 2.4 Total Anti‐RBD antibodies (Roche) SARS‐CoV‐2 detection

The determination of total SARS‐CoV‐2 antibodies(IgA, IgM, or IgG) directed against the spike protein was analyzed by the Electro‐chemiluminescence immunoassay (ECLIA) double‐antigen sandwich method by using Elecsys® Anti‐SARS‐CoV‐2 S assay. The Elecsys® Anti‐SARS‐CoV‐2 S immunoassay is a quantitative ECLIA that detects high‐affinity antibodies to the SARS‐CoV‐2 S protein receptor binding domain (RBD) and has a low risk of detecting weakly cross‐reactive and unspecific antibodies. The analyses were performed in accordance with the manufacturer's recommendations Elecsys® Anti‐SARS‐CoV‐2 S (Roche Diagnostics, GmbH) on COBAS e 411 immunoassay analyser.

Results are automatically reported as the analyte concentration of each sample in U/mL, with <0.80 U/mL interpreted as negative for anti‐SARS‐CoV‐2 S antibodies and ≥0.80 U/mL interpreted as positive for anti‐SARS‐CoV‐2 S antibodies. The sensitivity reported by the manufacturer was 98.1‐100% more than 28 Days post polymerase chain reaction (PCR) confirmation and an overall specificity of 99.98%.^22^

### 2.5 Anti‐RBD SARS‐CoV‐2 antibodies testing (Abbott)

The detection of anti‐SARS‐CoV‐2 antibodies against RBD was performed according to the recommendations of the manufacturer Abbott (Abbott Diagnostics, CH). The SARS‐CoV‐2 IgG II Quant assay is an automated, two‐step chemiluminescent microparticle immunoassay (CMIA). It is used for the qualitative and quantitative determination of IgG antibodies to the RBD of the S1 subunit of the spike protein of SARS‐CoV‐2 in human serum and plasma on the Alinity i system platform.

The Alinity workflow requires a minimum of 100 μL of serum or plasma. Sample, SARS‐CoV‐2 antigen coated paramagnetic microparticles, and assay diluent are combined and incubated. The IgG antibodies to SARS‐CoV‐2 present in the sample bind to the SARS‐CoV‐2 antigen coated microparticles. Anti‐human IgG acridinium‐labeled conjugate is added to create a reaction mixture and incubated. Following a wash cycle, Pre‐Trigger and Trigger Solutions are added. The resulting chemiluminescent reaction is measured as a relative light unit (RLU). There is a direct relationship between the amount of IgG antibodies to SARS‐CoV‐2 in the sample and the RLU detected by the system optics. The assay utilizes a 4 Parameter Logistic Curve fit data reduction method (4PLC, Y‐weighted) to generate a calibration and results. The result unit for the SARS‐CoV‐2 IgG II assay is AU/mL and the cut‐off is 50.0 AU/mL. The sensitivity and specificity reported by the manufacturer were 99.5% and 99.8%, respectively.^23^

### 2.6 VSV pseudo‐neutralization test for the detection of anti‐spike antibodies

Epithelial cell line Vero E6 (African green monkey kidney cells; CRL‐1586™. ATCC, 10801 University Boulevard), derived from the kidney of African green monkey Cercopithecus aethiops, were cultured in Dulbecco's modified Eagle's medium (DMEM) supplemented with 10% fetal bovine serum (FBS), antibiotic‐antimycotic mixture, Hepes buffer, l‐glutamine, and gentamicin solution. The cells were incubated at 37°C in a humidified atmosphere of 5% CO_2_. Replication‐competent VSV‐pseudovirus expressing the SARS‐CoV‐2 spike protein,^24^ (2019 n‐CoV/USA_WA1/2020) (a kind gift from J. Hiscott, *Istituto Pasteur Italia‐Fondazione Cenci Bolognetti*, Rome) was propagated in VeroE6 cells and harvested after cytopathic effects appearance at 72 h. Virus titer was determined using the Reed and Muench method^25^ and expressed as 50% tissue culture infective dose (TCID_50_/mL).

In vitro neutralization assay was performed to assess the extent to which antibodies are able to neutralize the replication of a competent VSV‐pseudovirus expressing the SARS‐CoV‐2 spike protein (Spike‐VSV) VeroE6 cells. Firstly, heat inactivation of plasma samples at 56°C for 30 min was carried out. Twofold serial dilutions (starting from 1:10) of plasma were prepared in empty, sterile 96‐well cell culture plates using culture medium (DMEM w/o FBS) and then mixed with an equal volume of viral solution containing 10^2^ TCID_50_/100 µL of Spike‐VSV (2019 n‐CoV/USA_WA1/2020). The plasma‐virus mixture was incubated 1 h at 37°C in a humidified atmosphere with 5% CO_2_. After incubation, 100 µL of each dilution mixture were transferred to duplicate monolayers of VeroE6 cells, seeded the day before at 2.5 × 10^4^ cell/well in 96‐well microtiter plates, and incubated in a humidified incubator at 5% CO_2_ for 1 h. After the incubation time was over, the virus‐plasma content was removed and replaced with culture medium. Following 72 h of incubation in a 5% CO_2_ environment at 37°C, anti‐Spike neutralizing titer was considered as the highest dilution of plasma samples without evidence of CPE in VeroE6 cells.

### 2.7 Statistical analysis

All data were analyzed with SPSS.20. Odds ratio (OR) was used to assess and quantify the strength of the association between seroprevalence and socio‐demographic parameters. Pearson's *χ* ^2^ Test (with 95% confidence interval [CI]) was used for the statistical analysis. Pearson correlation coefficients were used for correlation analysis *r* − 1 and 1. A statistical level of ≤0.05 was considered statistically significant.

## 3 RESULTS

### 3.1 Participant characteristics and demographic data

For the immune‐comparative analysis, 260 samples of the overall epidemiological survey carried out in a previous study,^21^ were selected and analyzed using CLIA (Roche), ECLIA (Roche), and CMIA (Abbott) and confirmed with the anti‐Spike pseudotyped VSV assay. The average age of the participants was 34.3 ± 12.9 years, ranging from 10 to 86 years, female gender represented 52.7% (137/260), for a male/female ratio of 1.1.

**Figure 2:**
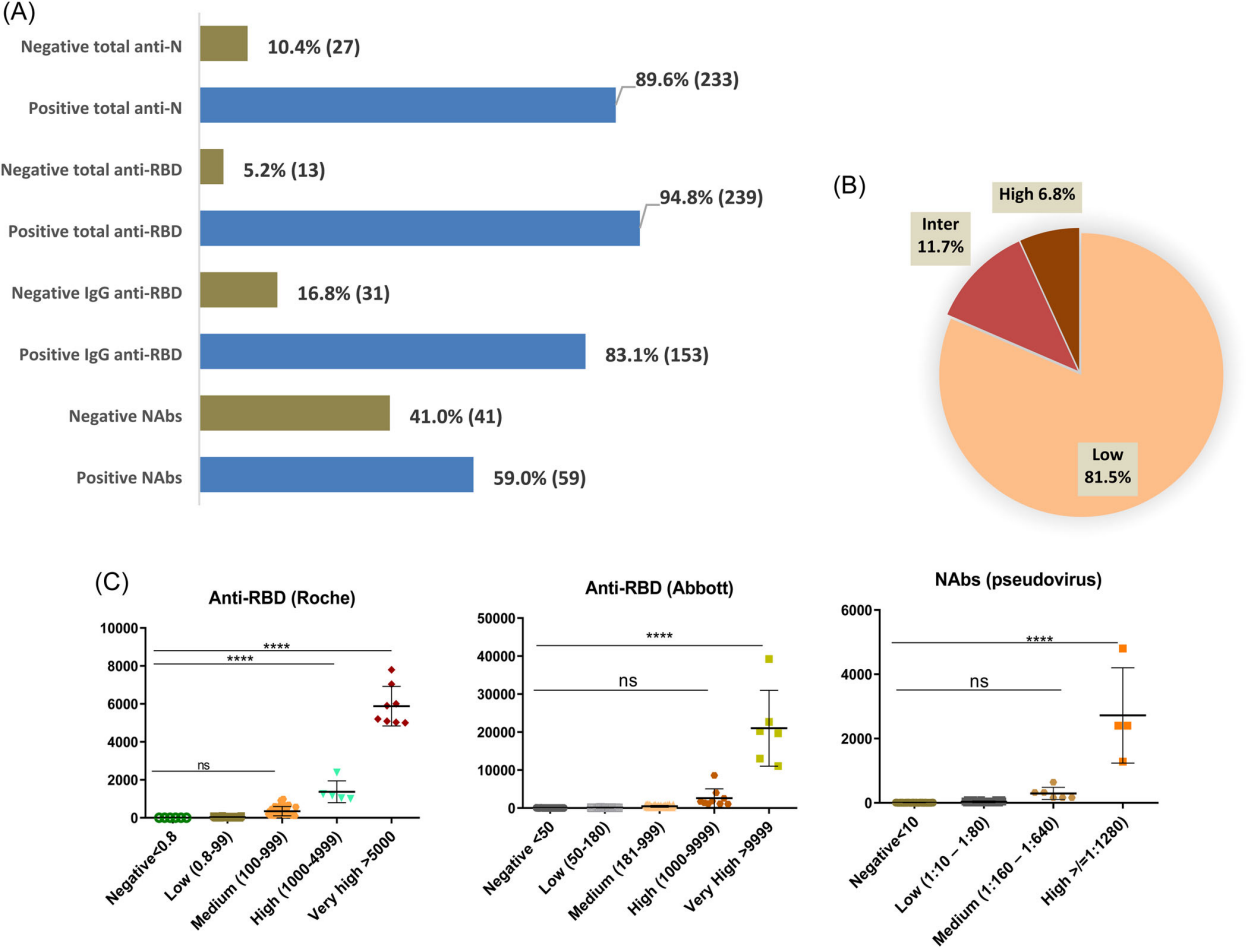
Participant characteristics and antibody classifications by method. (A) Antibodies positivity by method, (B) anti‐Spike neutralizing antibodies positivity range, and (C) antibodies range by method. Anti‐RBD, anti‐receptor‐binding domain antibodies; High Pos, high positivity (≥ 1:1280); inter Pos, intermediate positivity: (1:160‐1:640); Low Pos, low positivity (1:10‐1:80); NAbs, neutralizing antibodies.

Data indicated 89.6% (*n* = 233/260) samples positive for ant‐N and 94.8% (*n* = 239/252) for total anti‐RBD used Roche kit and 83.1% (*n* = 153/184) for IgG anti‐RBD used Abbott kit (Figure 2A).

88.5% (*n* = 223/252) of the samples were positive for both total anti‐N and total anti‐RBD antibodies. Moreover, 67.9% (*n* = 125/184) had both IgG anti‐RBD (Abbott) and total anti‐RBD (Roche), while 66.3% (*n* = 122/184) had both anti‐RBD and anti‐N antibodies (Table 1).

**Table 1:** Crosstable of different antibodies detected.

% (*N*)		Total anti‐RBD (ECLIA) Roche		IgG anti‐RBD (CMIA) Abbott	
		Negative	Positive	Negative	Positive
Total anti‐N	Negative	4.4 (11)	6.3 (16)	6.9 (11)	5.1 (8)
	Positive	0.7 (2)	88.5 (223)	10.7 (17)	66.3 (122)
Total Anti‐RBD	Negative	‐	‐	5.9 (9)	1.3 (2)
	Positive	‐	‐	12.4 (28)	67.9 (125)

Abbreviations: Anti‐RBD, anti‐receptor‐binding domain antibodies; CLIA, chemiluminescence immunoassay; CMIA, chemiluminescent microparticle immunoassay; ECLIA, electro‐chemiluminescence immunoassay; NAbs, neutralizing antibodies; VSV, vesicular stomatitis virus.

### 3.2 Detection of anti‐spike Nabs (VSV) and correlation with anti‐RDB antibodies levels

Of the 100 selected samples 59 (59.0%) resulted positive in the presence of NAbs using pseudotype test (Figure 2B). The positivity was confirmed both with anti‐RBD IgG (Abbott) test and total anti‐RBD (Roche). For the remaining 41 negative samples, 6 were detected negative in all the test used, 18 negatives using IgG anti RBD (Abbott) while 23 out the 41 negative Nabs sample were detected positive using both the anti‐RBD Abbott and Roche assay (Table 2).

**Table 2:** Cross report anti‐RBD and Neutralizing antibodies detection.

		VSV NAbs			
% (*N*)		Negative	Positive	*p* Value	***κ***
Total anti‐RBD (Roche)	Negative	14.6 (6)	0	0.004	0.2
	Positive	85.4 (35)	59.0 (59)		
IgG anti‐RBD (Abbott)	Negative	43.9 (18)	0	0.000	0.5
	Positive	56.1 (23)	59.0 (59)		

Abbreviations: Anti‐RBD, anti‐ receptor‐binding domain antibodies; NAbs: neutralizing antibodies, VSV, vesicular stomatitis virus.

Low NAbs levels (Titer range: 1:10–1:80) were found in 81.4% of anti‐spike NAbs positive plasma samples (*n* = 48/59). Seven samples (11.9%, *n* = 7/59) had intermediate NAbs levels (Titer range: 1:160–1:640) and only four patients (6.8%, *n* = 4/59) showed a high NAbs titers ≥1:1280 (Figure 2B).

Classified by category, the concentrations of total antibodies directed against RBD protein with Roche Cobas 411 indicated a significant difference with a *p* value < .0001, between concentrations ≥1000 U/mL and medium (100–999), low (0.8–99) or negative sample. Whereas Abbott Alinity I had revealed a significant difference *p* value < .0001 only for higher concentrations ≥9999 AU/mL similarly for the VSV spike neutralization assay ≥1:1280 (Figure 2C).

**Figure 3:**
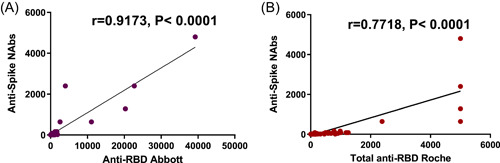
Correlation between anti‐spike neutralizing antibodies and anti‐RBD. (A) Correlation between neutralizing antibodies and anti‐RBD (IgG) with Abbott kit method and (B) correlation between neutralizing antibodies and total (IgA,M,G) anti‐RBD using Roche kit method, Anti‐RBD, anti‐receptor‐binding domain antibodies; Anti‐Spike NAbs, anti‐spike neutralizing antibodies; Anti total RBD, IgG, IgM, IgA antibodies against SARS‐CoV‐2 receptor binding domain; *r*, correlation coefficient.

Comparison of different methods used to detect antibodies against the spike protein revealed that all plasma samples positive to anti‐spike‐VSV NAbs were also positive for anti‐RBD with Abbott method, measured with chemiluminescent microparticle immunoassay. In accordance, a positive correlation was found between anti‐spike‐VSV NAbs titers and IgG anti‐RBD SARS‐CoV‐2 levels (Spearman's *ρ* coefficient, *r* = .9173, *p* < .0001) (Figure 3A). Similarly, all samples with NAbs to spike‐VSV showed the simultaneous presence of total anti‐RBD antibodies with Roche, detected with the electro‐chemiluminescence immunoassay (Spearman's *ρ* coefficient, *r* = .7718, *p* < .0001) (Figure 3B).

### 3.3 Agreement between SARS‐CoV‐2 NAbs and routine serological assays

**Figure 4:**
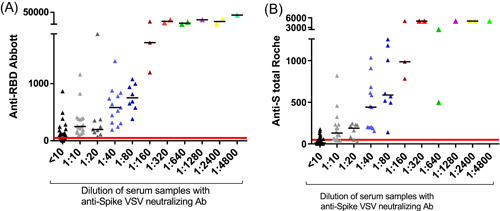
Quantitative correspondence between Anti‐RBD and neutralizing antibodies detection. (A) Quantitative correspondence neutralizing antibodies and anti‐RBD (IgG) with Abbott kit method and (B) correlation between neutralizing antibodies and total (IgA, M, G) anti‐RBD using Roche kit method. Ab, antibodies; Anti‐RBD, anti‐receptor‐binding domain antibodies; Anti‐S total, IgG, IgM, IgA antibodies against SARS‐CoV‐2 receptor binding domain; VSV, vesicular stomatitis virus.

The detection of NAbs, the threshold was considered positive for a titers ≥1:10 with the VSV method while it was ≥50 AU/mL with Abbott. The correlation between the positivity using VSV assay was strictly connected with the value obtained in anti IgG RBD Abbott test. In addition, all samples titers >1000 AU/mL showed positivity for NAbs detection (Figure 4A).

For the detection of total anti‐SARS‐CoV‐2 S antibodies with Roche Diagnostics method, 0.8 U/mL was used as the threshold for positivity. The results indicated that all samples positive with the VSV method were also positive with the Roche method. 35.0% (35) of positive samples between 0.8 and 175 U/mL were negative for NAbs, however, all samples titer >400 U/mL were positive for NAbs (Figure 4B).

Taken together, the Roche and Abbott methods showed agreement at high concentrations of antibodies with the pseudovirus method. However, *κ* agreement indicated a slight global agreement (*κ* value = 0.2, *p* = .000) between Roche and the VSV pseudovirus method, while a moderate global agreement (*κ* value = 0.5, *p* = .000) was observed between Abbott and the VSV method (Table 2).

### 3.4 Specificity and sensitivity of anti‐RBD (Abbott and Roche)

**Figure 5:**
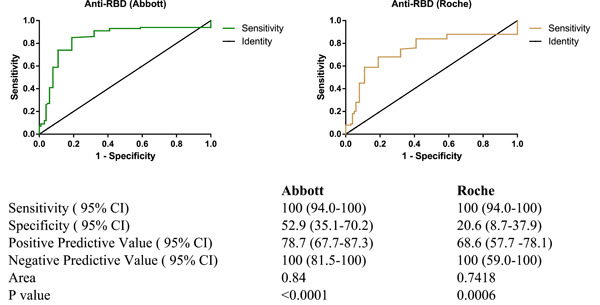
Comparison of anti‐RBD and neutralizing antibodies detection. Anti‐RBD (Abbott), anti‐receptor‐binding domain antibodies with Abbott kit method; Anti‐RBD (Roche), anti‐receptor‐binding domain antibodies with Roche kit method; CI, confidence interval.

Using the pseudotyped VSV neutralizing assay as the gold standard, our study indicated that both tests anti‐RBD (Abbott and Roche) showed a high sensitivity 100% (95% CI: 94.0–100), however, the Abbott Alinity i system test indicated a higher specificity (52% [95% CI: 35.1–70.2]) compared with the Roche cobas e 411 Elecsys® Anti‐SARS‐CoV‐2 S test which had a lower specificity (20.6% [95% CI: 8.7–37.9]) (Figure 5).

## 4 DISCUSSION

Immune memory against SARS‐CoV‐2 determines protection against reinfection, disease risk and vaccine efficacy. The interaction between RBD and the host cell receptor, ACE2, is responsible for the infection of host cells by SARS‐CoV‐2. The mechanism of NAbs is to block the receptor binding site between RBD and ACE2, thereby preventing infection of cells by SARS‐CoV‐2.^26^ Therefore, antibodies to the protein (NAbs) are thought to play a central role in host defence against SARS‐CoV‐2 infection and are therefore considered essential for recovery and protection against the viral disease.^27^, ^28^ It has been shown that most patients infected with SARS‐CoV‐2 develop antibodies that target the viral spike protein, as do individuals with the asymptomatic form.^28^, ^29^ The neutralizing capacity of anti‐SARS‐CoV‐2 antibodies is important as it represents a true protective immunity. The results of the study confirm that 59% of the samples, compared with all the tests, results to be positive for NAbs. This data support the asymptomatic diffusion of the virus observed in the study population in Chad.

In a general population based study including asymptomatic and patients, Dwyer et al. found a positive correlation between anti‐S and anti‐RBD antibodies and inhibition of ACE2 binding.^30^

We found a positive correlation between anti‐RBD and the detection of NAbs detection. Several groups have reported correlations between NAb titers to SARS‐CoV‐2 and various anti‐RBD or anti‐S IgG (or total IgG) assay titers.^31^, ^32^ Wajnberg et al. found that the vast majority of individuals infected with mild to moderate Covid‐19 have robust immunoglobulin G responses against the viral spike protein, with relatively stable titers, and that anti‐spike binding titers correlate significantly with neutralization of authentic SARS‐CoV‐2.^33^ According to these authors, these correlations were based on a specific level of antibody acquired by vaccination or natural infection that significantly reduces the risk of (re)infection. Therefore, suggesting that a simple determination of anti‐S and anti‐RBD IgG antibodies determination could be valuable in predicting the antibodies with ability to inhibit ACE2 binding in any individual.^30^

Using the pseudotyped VSV assay as the gold standard, our finding indicated that both the Roche Elecsys and Abbott serological tests (Anti‐RBD‐Abbott) for the detection of NAbs against skype protein had exceptionally good sensitivity 100% (95% CI: 94.0–100). These samples were collected between 12 August and 26 October 2021, about 3 months after the second wave of Covid‐19 in Chad.^1^ Referring to the validation data from Roche Diagnostics International Ltd, CH‐6343 Rotkreuz Switzerland, they describe a sensitivity of 100% within 28 days of a positive RT‐PCR and overall, a sensitivity of 98.8% (95% CI: 98.1%–99.3%). Like suggest by Torres et al., the high levels of maintenance of detectable NAb titers identified by the Roche Elecsys and Abbott serological tests (Anti‐RBD‐Abbott) are encouraging and provide further evidence of sustained protection against SARS‐CoV‐2 after natural infection.^34^ This means that both assays could be readily used to screen for anti‐RBD antibodies in an asymptotic population.

While the sensitivity of these two commercial tests was very satisfactory in our study context, the same cannot be said for specificity since we obtained a specificity of 52% with Abbott serological tests and 20.6% with Roche Elecsys. Although our results are apparently in contrast with those previously published^35^, ^36^ that have reported higher specificity values it should be considered such differences can be due to the inclusion criteria of the analyzed cohort. Indeed, differently with others we focused the study only on asymptomatic samples collected during Covid‐19 pandemic.

### 4.1 Samples distribution across different test methods

We notify here that the samples tested were not equally distributed among all different tests used during the study. This could be considered as a limitation of the study even though our study was not designed for different tests performance comparison at the beginning.

Among other limitations of this study were the unavailability of serum from groups of confirmed positive patients during sample collection, follow‐up, symptomatic samples and pre‐pandemic samples which would have allowed a better evaluation of these commercial tests as well as the kinetics of these antibodies. The level of long‐term persistence of NAbs requires further study and a larger sample size.

### 4.2 Limitations of the study

The main limitation of this study is the difference in the number of individuals tested by different methodologies, due mainly to the availability of biological samples. Due to the tests cost and the need of an adequate technical platform for pseudotype analysis only a limited number of samples have been confirmed with all the test.

However, this study, made it possible to identify tests with specificity, sensitivity, and cost most suited to the context. Although the Covid‐19 situation has been stable for several months in Chad, which is one of the Central African countries with the lowest number of cases and deaths due to Covid‐19,^1^ data on the variants currently in circulation would improve the quality of this study.

## 5 CONCLUSION

Finally, our results indicated a high presence of NAbs against the SARS‐CoV‐2 spike protein among asymptomatic populations in the city of N'Djamena. Considering the high seroprevalence (69.5%) of anti‐N obtained in 2700 asymptomatic volunteer donors in the first phase of the study and the results of this cohort, it is clear that this asymptomatic population in the city of N'djamena has the capacity to produce different types of anti‐SARS‐CoV‐2 antibodies (anti‐N, anti‐S, NAbs) following contact with this virus.

Humoral immune protection could be one of the reasons for the low prevalence and low severity of Covid‐19 observed in this locality, as these NAbs are known to play a key role in blocking SARS‐CoV‐2 infection. Moreover, the commercial tests available (Abbott Alinity i and Roche Elecsys®) for the detection of these anti‐RBDs showed acceptable performance when compared with the pseudovirus neutralization test.

## AUTHOR CONTRIBUTIONS

Mahamat Fayiz Abakar, Guy Rodrigue Takoudjou Dzomo, Ali Mahamat Moussa, and Ali Mahamat Moussa contributed to the study design, protocol validation. Andrillene Laure Deutou Wondeu drafted the manuscript. Federica Frasca, Giulia Linardos, Fatima Abdelrazakh and Andrillene Laure Deutou Wondeu performed laboratory analysis. Nathan Naibei conceived and supervised the electronic data collection. Andrillene Laure Deutou Wondeu, Nathan Naibei, and Mahamat Fayiz Abakar conducted statistical analysis. Carolina Scagnolari, Guido Antonelli, Cristina Russo, Carlo Federico Perno, John Hiscott, Vittorio Colizzi, and Giulia Cappelli coordinated the fieldwork. Vittorio Colizzi and Giulia Cappelli contributed with comments and final edits. All authors read and approved the final version of the manuscript.

## CONFLICT OF INTEREST STATEMENT

The authors declare no conflict of interest.

## ETHICS STATEMENT

The present study was approved by the National Bioethics Committee in Chad (*Comité national de bioéthiques du Tchad* “CNBT” Decision: N°004/PR/MESRI/SE/DGM/CNBT/SG/2022). Informed consent was obtained from all study participants before blood sampling. It was stated that participation is voluntary, without compensation and that individuals could withdraw from the study at any time, without negative consequences for them, their family or their community.

## Data Availability

All data generated or analyzed during this study are included in this article.
